# Fluid Viscosity Measurement by Means of Secondary Flow in a Curved Channel

**DOI:** 10.3390/mi13091452

**Published:** 2022-09-02

**Authors:** Maxim I. Pryazhnikov, Anton S. Yakimov, Ivan A. Denisov, Andrey I. Pryazhnikov, Andrey V. Minakov, Peter I. Belobrov

**Affiliations:** 1 Laboratory of Physical and Chemical Technologies for the Development of Hard-to-Recover Hydrocarbon Reserves, Siberian Federal University, 660041 Krasnoyarsk, Russia; 2 Laboratory of Heat Exchange Control in Phase and Chemical Transformations, Kutateladze Institute of Thermophysics, 630090 Novosibirsk, Russia; 3 Department of Biophysics, Siberian Federal University, 660041 Krasnoyarsk, Russia

**Keywords:** microfluidics, viscometer, viscosity, curved channel, secondary flow, flow recirculation, glycerol

## Abstract

This article presents a new approach to determining the viscosity of Newtonian fluid. The approach is based on the analysis of the secondary Dean flow in a curved channel. The study of the flow patterns of water and aqueous solutions of glycerin in a microfluidic chip with a U-microchannel was carried out. The advantages of a microfluidic viscometer based on a secondary Dean flow are its simplicity, quickness, and high accuracy in determining the viscosity coefficient of a liquid. A viscosity image in a short movie represents fluid properties. It is revealed that the viscosity coefficient can be determined by the dependence of the recirculation angle of the secondary Dean flow. The article provides a correlation between the Dean number and the flow recirculation angle. The results of the field experiment, presented in the article, correlate with the data obtained using computational fluid dynamics and allow for selecting parameters to create microfluidic viscometers with a U-shaped microchannel.

## 1. Introduction

Viscosity is a key parameter influencing flow and an important macroscopic characteristic of solutions. Currently, mechanical macrorheometers based on flow resistance are well designed. They use elements with different geometries (coaxial cylinders, cone and plate combination, and parallel planes) that drive the studied liquid, whose volume is just several milliliters. One of the problems of existing mechanical macrorheometers is associated with the minimum measurable torque, which leads to an increase in the error when determining the viscosity while decreasing the shear rates. For biomedical measurements, it is often necessary to analyze liquid samples of a microliter volume, while it is important to not allow contamination and to save the sample for further measurements of other properties. All of this led to the development of microfluidic viscometers [1,2]. The design of microfluidic viscometers differs from macroscopic ones by the absence of mechanical movable components and a smaller number of required technological operations during their production.

There are various operation principles of existing microfluidic devices for rheology analysis. Most microfluidic devices assume a visual assessment of capillary effects [3,4] using the analysis of images from a camera [5], including images from a smartphone camera [6]. In terms of determining the characteristics of liquids, drip-measuring systems [7,8,9] are more successful; however, they have a more complex design and an optical signal registration system. There are viscometers based on measuring the liquid velocity when flowing in capillaries [10,11] or through micro-membranes [12]. The viscosity can be determined by the pressure drop in the channel during static movement [5,13,14,15,16,17,18,19] or pulsed flow [20]. Existing microfluidic chips with an obstacle in a hyperbolic channel allow for creating compression–expansion loads to characterize viscoelastic effects in the flow at high velocities [21]. Particle migration analysis is also used to study the rheology of the viscoelastic suspension flow in microchannels [22,23]. In addition, certain methods allow for determining the viscosity indirectly by the deformation of an elastic chip under pressure [24]. Jang et al. [25] proposed a viscosity measurement method that utilizes the flow characteristics of vertical paper-based fast-flow. Other examples of microfluidic viscometers and their pros and cons can be found in [26,27]. The main disadvantages of existing microfluidic viscometers are the complex fabrication of microfluidic devices, the requirement for two or more reference fluids (or particle trackers), and the inability to integrate into a lab-on-a-chip and μ-TAS.

In our work, an optical microfluidic viscometer based on secondary flow in curved channels was proposed. In curved channels, the fluid flow is subjected to centrifugal force. As a result, the point of maximum velocity distribution is shifted from the center of the channel to the concave wall of the channel. This shift results in a sharp velocity gradient between the point of maximum velocity and the concave wall. A sharp velocity gradient causes an increase in pressure, and the local velocity near the walls is not enough to fully balance this pressure gradient. This imbalance leads to recirculation of the fluid flow in the form of vortices (known as Dean vortices [28,29]) directed from the center of the channel to the outer wall of the channel and back to the center to balance the pressure gradient. The characteristics of the observed vortices depend on the rheological properties of the liquid.

In this article, we decided to conduct an in-depth study to test the relationship between the formation of secondary Dean flows in microfluidic chips and the viscosity of the liquid to use this relationship when evaluating the potential parameters of a microfluidic viscometer. The proposed viscometer would differ for the better from the existing microfluidic viscometers in that a reference liquid or expensive sensitive sensors are not required. Only a video camera and means for measuring the liquid flow rate (for example, a measuring container) are sufficient.

## 2. Materials and Methods

### 2.1. Description of the Approach

Studying flows in curved channels has been within the researchers’ focus for a long time, and became updated with the development of microfluidics [30,31]. Curved flows are actively used to create a variety of mixers [32] and devices providing particle focusing [33,34]. The influence of viscosity on secondary flows was mentioned earlier [35].

A curved rectangular microchannel with a width *w* and a height *h* was considered. The flow in such a microchannel was determined by the Reynolds number and the Dean number. The Reynolds number is the ratio of inertia forces to viscous forces:

(1) $$Re=\frac{\rho UD}{\mu },$$

where ρ is the liquid density; U=Q/A is the characteristic velocity (equal to the ratio of the average volumetric flow *Q* to the cross-sectional area of the microchannel *A*; *D* is the hydraulic diameter; μ is the dynamic viscosity. The hydraulic diameter of the microchannel is defined as:

(2) $$D=\frac{4A}{P},$$

where A=h·w is the cross-sectional area of the microchannel; P=2(h+w) is the perimeter of the microchannel cross-section.

The Dean number represents the ratio of the transverse fluid flow arising from the curvature of the channel to the longitudinal flow:

(3) $$De=Re\sqrt{\frac{D}{2r}},$$

where *r* is the radius of the channel curvature.

Substituting (1) into (3), and expressing the viscosity μ, we obtained the following expression:

(4) $$\mu =\frac{\rho UD}{De}\sqrt{\delta },$$

where δ=D/2r is the ratio of the channel curvature.

**Figure 1:**
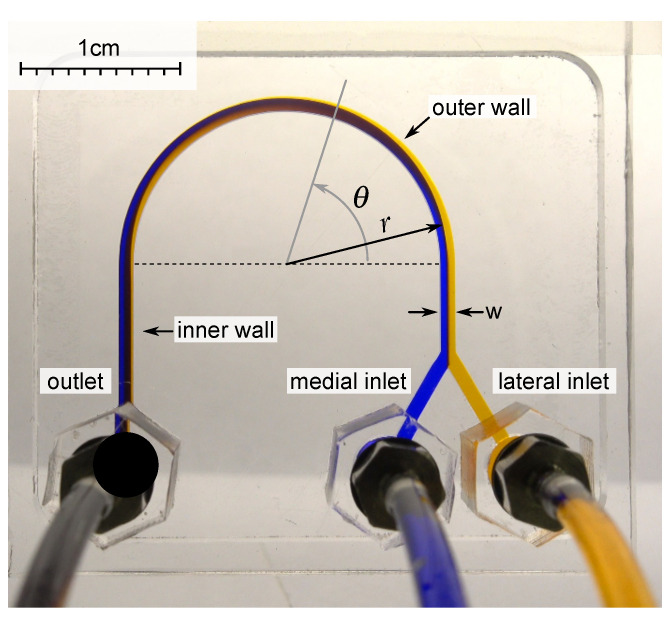
A microfluidic chip from PMMA with U-shaped microchannel with a width *w* = 920 μm, a height of 500 μm, and a radius of curvature *r* = 10 mm was used for experimental investigation. The liquid with two different dyes was pumped into the medial and lateral inlets with the same flow rate. The angle θ was measured from the start of curvature.

When a liquid flows in a curved channel, two opposing laminar recirculations appear in the channel, perpendicular to the flow direction. While marking the liquid with dye in the lateral part of such a channel, one can observe that the flow will carry liquid to the medial part at a certain distance. At the same time, the pure liquid from the medial part will end up in the lateral part. During this process, mixing is insignificant. The angular distance at which the flow is turned over is called the recirculation angle. In addition to the viscosity, the recirculation angle is also affected by the flow velocity (which is related to the flow rate and the hydrodynamic diameter of the channel) [36] (Figure 1).

The most compact and easy-to-use microfluidic measuring systems can be designed using the proposed approach. Such a viscometer is easier to integrate into existing microfluidic measuring systems [37,38]. The results presented in the article can act as a guide to the development and creation of curved microfluidic viscometers for a wide range of applications and soft matter studies.

### 2.2. Microfluidic Device Fabrication

The chip was made of poly(methyl methacrylate) (PMMA, Novattro, Russia) by milling [39,40] and solvent bonding [41].

Milling was performed using a milling machine Modela MDX-40a (Roland, Hamamatsu, Japan) with a single-tooth cutter with a polished cutting edge, one mm in diameter 0078310E (Datron AG, Mühltal, Germany) in a bath with distilled water. The cutting depth was 0.1 mm, the advance per tooth was 12 microns, and the cutting speed was 47.12 m/min. To glue the chip of PMMA, dichloroethane-1,2 was used. For this, the solvent was applied to the glued area by spraying, and the PMMA plates were pressed against each other under a pressure of 180 kPa for one minute. To connect the tubes to the chip, fittings M-3AU-2 (SMC, Tokyo, Japan) were used, which were screwed into nuts made of PMMA, previously glued with a flange to the inlet and outlet orifices of the chip (Figure 1).

### 2.3. The Tested Liquids

Distilled water, aqueous solutions of glycerin, 30% and 50% by weight, were used as test liquids.

The properties of test liquids are presented in Table 1. The viscosity coefficient was measured using a DV2T rotary viscometer (Ametek Brookfield, Middleboro, MA, USA) with a ULA adapter. The density was determined by the pycnometer method (the error of determination was 0.1%).

### 2.4. Experimental Setup and Procedure

The dimensions of the viscometer were preliminary estimated using numerical simulation. The geometric dimensions were selected in such a way as to measure the viscosity of water at a flow rate of 1 mL/min. Since we wanted to develop a small microfluidic device, we limited the radius of curvature to 1 cm. As a result of the assessment, the characteristic hydrodynamic size of the microchannel was approximately 700 μm. Thus, a microchannel device with a width of 920 μm and a height of 500 μm was manufactured (Figure 1). The chip was connected to Hamilton syringes (Gaslight, 1005 LT) using polyurethane tubes TU0212C-20 (SMC, Tokyo, Japan) with an ID of 1.2 mm (OD 2 mm). The liquid flow rate was set by the SPLab02 syringe pump (Baoding Shenchen Precision Pump Co., Baoding, China). The minimum volume of liquid required for one measurement is 150 μL. The volumetric flow rate *Q* in the U-microchannel varied from 0.1 to 20 mL/min (the accuracy of setting the flow rate was 0.3%). This corresponded to the Reynolds numbers ranging from 5 to 300, and the Dean numbers ranging from 1 to 55 for water. The flow patterns were recorded using a Sony RX100 IV camera, and their further analysis is presented below. After each experiment, the U-microchannel was thoroughly washed. A Meros TCU-100 temperature controller (Dolomite Microfluidics, Royston, UK) and a RE112 thermostat (Lauda, Lauda-Königshofen, Germany) were used. The measurements were carried out at a temperature of 25 ${}^{\circ }$C.

### 2.5. Computational Fluid Dynamics (CFD)

The numerical simulation was used for preliminary estimation of the hypothesis; understanding the three-dimensional flow pattern underlying the proposed method; as well as estimating the geometric dimensions of used microchannel.

### 2.6. Secondary Flow Quantification

**Figure 2:**
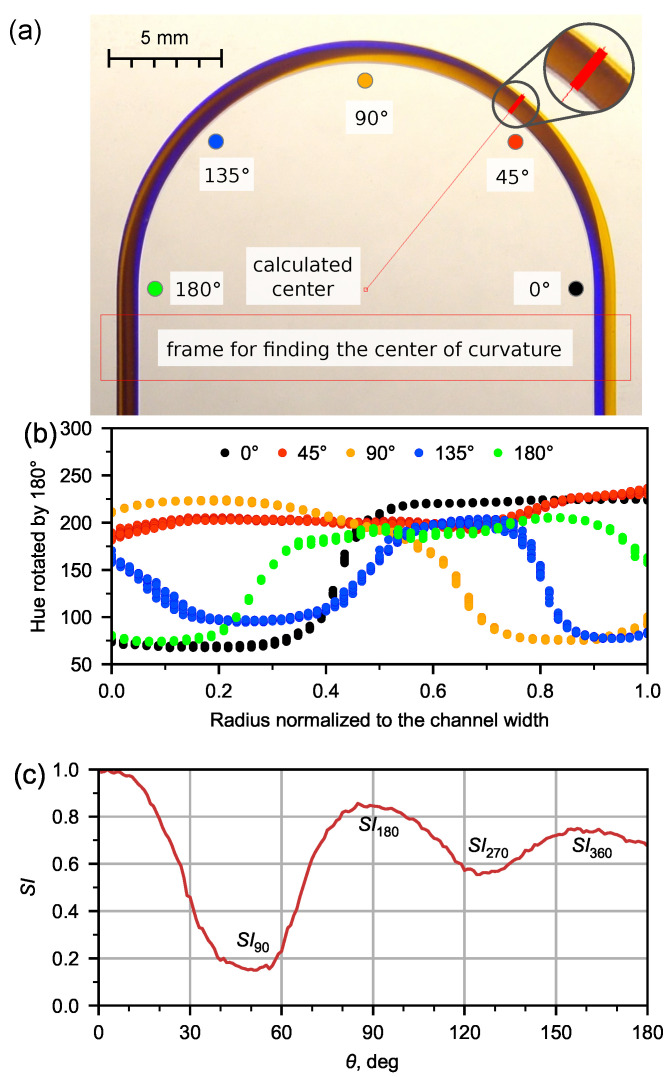
Analysis of experimental image of flow pattern for water at *Q* = 5 mL/min by means of developed application. (**a**) Automatic detection of curvature center and demonstration of selecting pixels set by the beam at fixed angle; (**b**) hue distribution in the channel in 5 various cross-sections; (**c**) dependence of switching index (SI, normalized standard deviation of selected pixels hue) on the angle θ with labeled extremes corresponding to various flow recirculation angles.

A quantitative assessment of the secondary flow was carried out by resembling method, as is described in work of Bayat and Rezai [35]. They determined switching index (SI), which is normalized standard deviation of intensity values for set of image pixels along beam cross section. An application was developed to analyze the secondary Dean flow based on obtained images. The application allows for the opening up of the image of the channels with tinted flows, determining the center of curvature by selecting the area where the inlet and outlet channels run parallel (Figure 2a), and performing a color analysis based on the hue, saturation, value (HSV) model after setting the pixel sampling parameters depending on the beam inclination. To develop the application, the BlackBox Component Builder component framework (Oberon Microsystems, Switzerland) and the FreeImage library were used. The application is available at https://github.com/iadenisov/imgs-analyzer (accessed on 27 May 2022).

The application could also automatically calculate the average value of the hue in the cross-section and derive the hue dispersion (5) in the microchannel depending on the angle at unit intervals (Figure 2b).

(5) $$\sigma^{2}=\frac{1}{180}\sum_{\theta =0}^{180}\left(H_{\theta }-\bar{H}\right)$$

In the present work, for the quantitative analysis of the secondary Dean flow, the switching index (SI) was used [35]:

(6) $$SI=\frac{\sigma }{\sigma_{max}}$$

## 3. Results and Discussion

### 3.1. Flow Patterns Analysis

We will describe in more detail the analysis of the secondary flow using the switching index on the example of an experimental flow pattern of water at 5 mL/min (Figure 2a). For each flow pattern obtained in the microfluidic chip, we determined the value of the hue (*H*) in the cross section of the microchannel (Figure 2b) obtained for the SI distribution along the length of the U-microchannel (Figure 2c). To find the extremes of the dependence SI(θ), an approximation was made by a polynomial of the second degree in the region of the extremes.

The switching index is maximal at the inlet, then decreases down the channel to a certain minimum at θ = 50.5${}^{\circ }$ (the red ray in Figure 2a corresponds to this angle), and then increases again.

**Figure 3:**
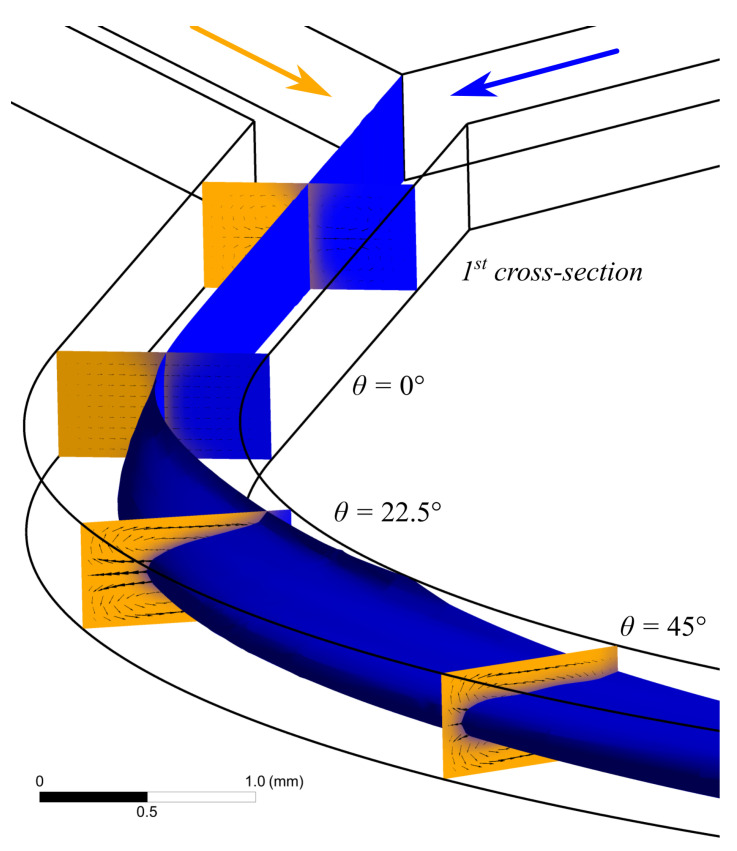
Results of computer simulation performed in application AnsysFluent illustrates appearance of secondary vortex in curved channel with water flow rate at 5 mL/min. Isosurface of the volumetric fraction of the liquid phase. The arrows show the velocity vector field. The flows at angle 50.5${}^{\circ }$ corresponding to the $SI_{90}$ are used for measuring viscosity.

A numerical simulation of the water flow in the U-microchannel was carried out. Visualization of a 3D water flow at a flow rate of 5 mL/min using the isosurfaces of the volumetric fraction of the liquid phase is shown in Figure 3. In addition, Figure 3 shows contours of the phase distribution and the velocity vector field in several cross-sections of the microchannel. After the Y-connection of the inlet sections (cross-section 1 in Figure 3), the boundary gets slightly blurred due to diffusion. The flow gets stable before the microchannel starts bending. This can be seen by the velocity vector field in the section θ = 0${}^{\circ }$. Further, two Dean vortices are formed along the channel (Figure 3). The recirculation index is minimal when the initially medial water flow (blue) is completely squeezed between the two initially lateral water flows (orange).

The recirculation angle θ = 89.4${}^{\circ }$ is responsible for flow recirculation by 180${}^{\circ }$ (Figure 2c). The liquid at the medial wall of the microchannel begins to flow along the lateral wall (Figure 2a). The same situation occurs with the liquid that initially flowed along the lateral wall and then moved to the medial one. This flow is responsible for the first maximum of the dependence SI(θ) (see Figure 2c). The second minimum and maximum of SI(θ) (Figure 2c) correspond to the recirculation of the flow by 270${}^{\circ }$ (θ = 126.6${}^{\circ }$) and 360${}^{\circ }$ (θ = 159.8${}^{\circ }$), respectively.

**Figure 4:**
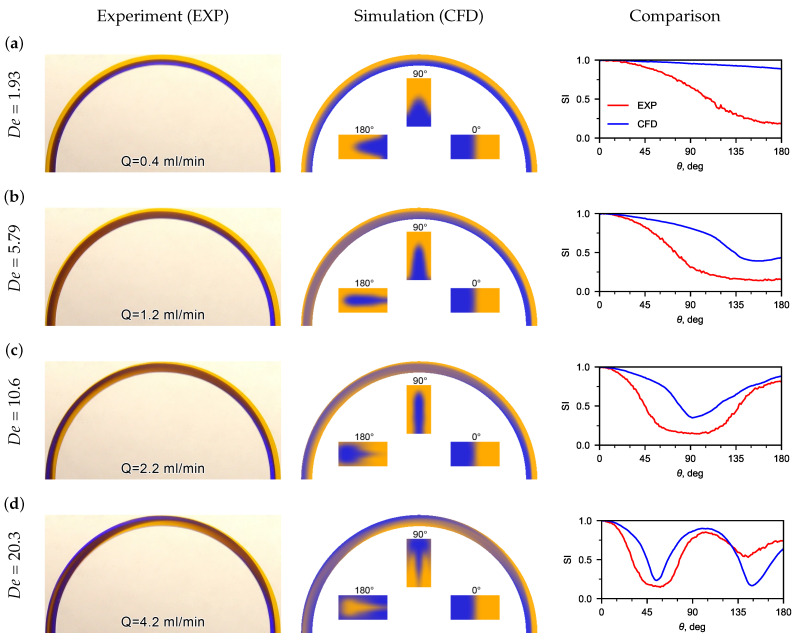
Experimental (left pictures) and calculated (CFD) (center pictures) flow patterns for water at different De numbers. On the right pictures are the distributions of SI depending on the angle θ (the red line is the experiment, the blue line is the CFD). The inserts in the calculated flow patterns are the phase distribution contours in sections 0${}^{\circ }$, 90${}^{\circ }$, and 180${}^{\circ }$, which clearly display the secondary Dean flow in the U-microchannel. (**a**) flow patterns for water at De = 1.93; (**b**) flow patterns for water at De = 5.79; (**c**) flow patterns for water at De = 10.6; (**d**) flow patterns for water at De = 20.3.

Experimental flow patterns were obtained, as well as a comparison with flow patterns obtained using CFD (Figure 4). The patterns of the mutual flow of tinted flows are affected by the liquid flow rate (Figure 4a). It was observed that the flow pattern was sensitive to the inlet flow velocity. Thus, at very low water flow rates (*Q* < 0.4 mL/min), no recirculation of the flow was detected in the U-microchannel (Figure 4a). The boundary of the tinted flows is clear and located near the middle of the microchannel width. This is clearly seen in the flow patterns and phase contours obtained using CFD.

When the water flow rate increases to *Q* = 1.2 mL/min, the boundary of the tinted flows shifts to the lateral wall, and an extremum ($SI_{90}$) appears on the dependence of the standard deviation of the hue from the microchannel turn angle (see Figure 4b). The recirculation angle for $SI_{90}$ is 159.3${}^{\circ }$. The recirculation angle corresponding to the switching index $SI_{90}$ decreased to 93.3${}^{\circ }$ with an increase in flow rate from 1.2 mL/min to 2.2 mL/min. A mutual recirculation of the flow of the medial and lateral flows (180${}^{\circ }$) is observed in the U-microchannel at a Dean number of about 11. A full recirculation of the flow (360${}^{\circ }$) can be observed when Dean numbers are greater than 20. This can be seen in the inserts of cross-sectional flow patterns obtained using CFD and the dependence of SI on θ (see Figure 4c).

The SI dependences, obtained from experimental photographs and calculated flow patterns, were compared along the U-microchannel length. A good matching of the switching index was obtained for recirculating the flow at 90${}^{\circ }$, 180${}^{\circ }$, 270${}^{\circ }$, and 360${}^{\circ }$.

Discrepancy between the experimental and CFD results for SI vs. theta plots (Figure 4) may be explained due to the fact that, in CFD, the color of the liquid at the inlet was set approximately to the color from experimental photos. However, it is important that the extremes of the dependencies coincide well enough.

**Figure 5:**
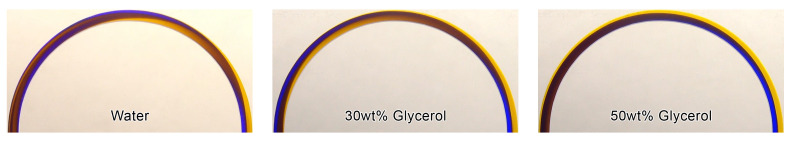
Experimental flow patterns of the water and aqueous solutions of glycerol at *Q* = 6 mL/min.

The flow patterns of aqueous solutions with different contents of glycerol at a fixed flow rate are different (Figure 5). An increase in the viscosity of an aqueous glycerol solution leads to the fact that a higher flow rate is required to obtain the same flow pattern as for water.

### 3.2. Determining Fluid Viscosity

**Figure 6:**
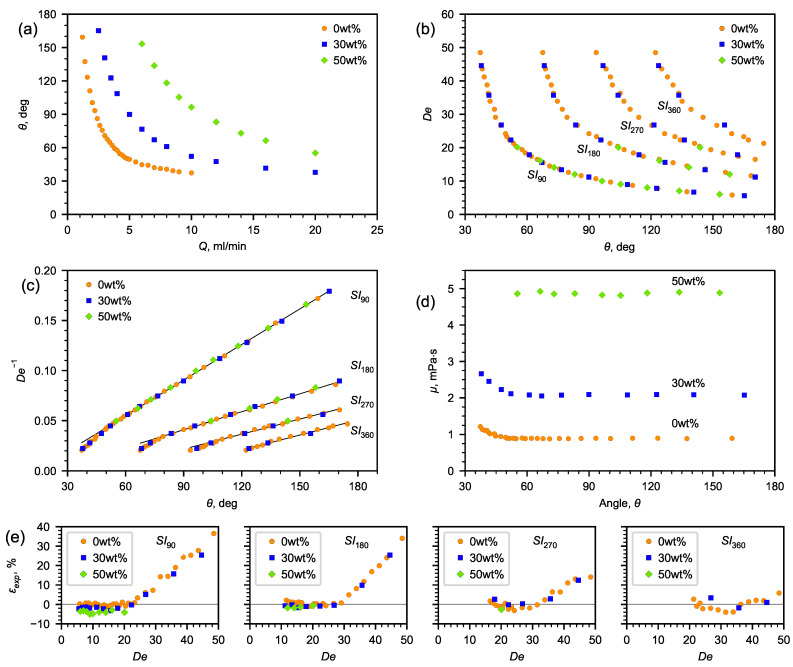
The results of experimental study. (**a**) Dependence of flow recirculation angle $SI_{90}$ on fluid flow rate; (**b**) Dean number vs. flow recirculation angle; (**c**) inverse Dean number vs. flow recirculation angle; (**d**) viscosity vs. flow recirculation angle; (**e**) relative deviation between the dynamic viscosity coefficient calculated according to (3) and the reference data [42] in the investigated range of the Dean number.

The obtained correlations of the angle depending on the liquid flow rate for 30 and 50% aqueous solutions of glycerol, which are presented in Figure 6a (the spread of the angle values does not exceed the size of the symbols), show that, as the flow rate increases, the angle decreases. With an increase in the viscosity of the liquid, the flow rates shifted towards high values of *Q*. However, building the dependence of the angle on the Dean number, one can observe a self-similarity of the flow pattern. Each De number corresponds to a certain flow pattern and a certain angle. According to (4), knowing the dependence of De on the recirculation angle and the flow rate corresponding to the obtained flow pattern, it is possible to determine the dynamic viscosity of the liquid (Figure 6d).

In order to obtain the dependence of De on the angle, $SI_{180}$, $SI_{270}$, and $SI_{360}$ were determined both for water and aqueous solutions of glycerol (Figure 6a).

Since the viscosity has an inverse relationship with De, the inverse Dean number is better suited for analysis (Figure 6b).

As is seen from Figure 6c, the experimental data can be approximated by a linear correlation (7):

(7) $$\frac{1}{De}=a\theta +b$$

where *a* and *b* are the fitting coefficients

When determining the fitting coefficients (Table 2), the analysis was carried out using the linear part of the experimental data (Figure 6c). Using it, angle θ was expressed in radians. Data corresponding to high flow rates and Dean numbers were not taken into account. Table 2 also contains the certainty factors, the average absolute deviations (AADs), and the maximum absolute deviations (MADs) between the experimental data and the approximations values. Table 2 also shows the ranges of the De numbers, within which, the fitting coefficients were determined, and the minimum angle, below which, the deviation of the data from the linear dependence increases.

The following characteristics reflecting the accuracy of determining the viscosity using the microfluidic method with a U-microchannel were established (Figure 6e). On the lower side is the accuracy of determining the rotational angle and the geometric limitations of the channel, whereas on the upper side is the nonlinearity of the dependence of the inverse De number on the rotational angle. There is reason to assume that the extension of the range to the lower side (at low velocities) is possible using curved channels, in which, the channel bend will tend to $360^{\circ }$, as well as employing spiral channels with a changing radius of curvature.

The analysis has shown that the De number can be determined with a high accuracy from the known dependence on the angle $SI_{90}$(θ).

Further, the dynamic viscosity coefficients of water and aqueous solutions were calculated according to Equation (8), taking into account the De number from (7) using the obtained coefficients *a* and *b* from Table 2.

(8) $$\mu =\left(a\theta +b\right)\frac{\rho QD}{A}\sqrt{\delta }$$

If the density is unknown, then, in Equation (8), it can be omitted by replacing the dynamic viscosity with the kinematic one:

(9) $$\nu =\left(a\theta +b\right)\frac{QD}{A}\sqrt{\delta }.$$

Figure 6e shows the deviation of the calculated viscosity from the reference data [42] within the entire studied range of the Dean numbers, as well as the ranges of the De number in which the viscosity deviation from the reference data is minimal. It was revealed that the average relative deviation for water was 0.8, 2.3, and 3.22%, for glycerol solutions—30% and 50%, respectively (Table 3). The minimum flow recirculation angles are determined, below which, the error of determining the viscosity coefficient by the proposed microfluidic viscometer increases. A relatively small change in the flow rate at its high absolute values has a weak effect on the change in the flow recirculation angles. Conversely, a greater sensitivity in changing the flow recirculation angle is observed at a small change in low flow rates.

Table 3 presents the viscosity coefficients obtained using the developed microfluidic viscometer ($\mu_{exp}$), obtained using the rotational method ($\mu_{rot}$), as well as the data retrieved from the literature ($\mu_{ref}$) [42]. The accuracy of determining the viscosity coefficient using a microfluidic viscometer ($\varepsilon_{exp}$) is no worse than that measured by a rotary viscometer ($\varepsilon_{rot}$).

### 3.3. Methodology for Design of Microfluidic Viscometer

The microfluidic device proposed in the present work can be designed for the required range of measured viscosity values. It is possible to measure equally low-viscosity (water-like) and theoretically high-viscosity liquids with a higher curvature and lower hydraulic diameter.

The offered viscometer is able to be calibrated for liquids of known viscosity (for example, water). For this, the switching index is determined at different flow rates. Then, the correlation coefficients can be determined (7). After that, the dynamic viscosity of the test fluid can be measured according to Equation (8).

The procedure for determining the viscosity coefficient of an unknown fluid is as follows. 1. First, the density of the unknown fluid must be measured. 2. Then, the recirculation angle must be determined at a fixed flow rate. 3. The recirculation angle at different flow rates is determined. 4. The viscosity coefficient is determined μ according to Formula (8), substituting the geometric dimensions, sample density, flow rates, and recirculation angles. 5. The result is averaged.

Moreover, the advantage of the proposed microfluidic viscometer is that, if the density is unknown, then the kinematic viscosity can be estimated instead of dynamic viscosity:

(10) $$\nu =\frac{Q}{De}\xi ,$$

where $\xi =\frac{D}{A}\sqrt{\delta }$ is the geometric complex of the U-microchannel. For the geometry studied in the present research, ξ=260.

**Figure 7:**
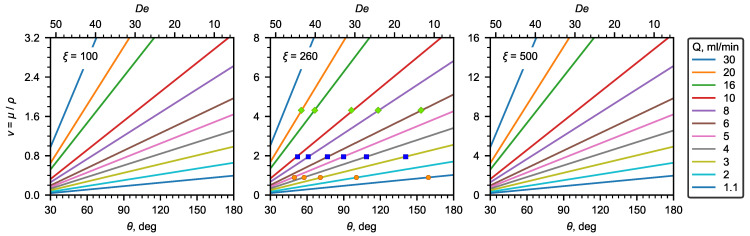
Nomograms of kinematic viscosity at different ξ.

As can be seen, the kinematic viscosity depends linearly on the recirculation angle and the liquid flow rate. Figure 7 shows a nomograph for determining the kinematic viscosity using the recirculation angle $SI_{90}$ (De number) and liquid flow rates for the U-microchannel geometry under study. In addition, the experimental data obtained for water and aqueous solutions of glycerol are plotted in the same Figure 7.

In this work, the effect of the geometric dimensions (width, height, and radius of curvature) of the U-microchannel was not considered. Therefore, for other geometric parameters of the microchannel, the correlations obtained in this work should be used with care. Below, we give some estimates for selecting geometric parameters to choose the values that are most optimal for determining viscosity. For low-viscosity liquids, the optimal geometry will be the one with a relatively small ξ (∼100–300).

Thus, for the U-microchannel with a square cross-section, one mm in diameter, and a radius of curvature of the channel of 5 cm, ξ=100. In general, ξ depends on the diameter and radius of curvature according to the following relation:

(11) $$\xi =\frac{1}{\sqrt{2Dr}}$$

As for the estimated range of flow rates that allow for observing the secondary flow in a U-viscometer, De numbers, which allow for determining the viscosity well, are within a range from 5 to 20 (Table 2). The upper limit of the De number is associated with the deviation of the dependence from the linear one at the high velocities of the main flow. As already noted above, at low De numbers, no flow recirculation was observed. In addition, high requirements are imposed on the accuracy of the algorithm for determining the extremum point of the SI(θ) dependence for calculating the flow recirculation angle (Figure 4a, De=5.8). This defines the lower limit of the range of the De numbers. Expressing the flow rate from (10) and substituting the De number, we obtain the minimum $Q_{min}$ and maximum $Q_{max}$ flow rates (mL/min):

(12) $$Q_{min}=0.03\cdot \nu \cdot \sqrt{2dr},$$

(13) $$Q_{max}=0.12\cdot \nu \cdot \sqrt{2dr},$$

where the quantities have the following physical dimensions: ν—(mm${}^{2}/$s), *d*—(μm), *r*—(cm).

## 4. Conclusions

In this article, a study of the secondary Dean flow of the Newtonian fluid in the U-microchannel was conducted to determine liquid viscosity. A tool for analyzing and determining the recirculation angle of the flow based on the secondary Dean flow patterns was developed, and an empirical dependence of the viscosity coefficient of Newtonian liquids on the flow recirculation angle was obtained. The study was conducted for water and aqueous solutions of glycerin. It was shown that the viscosity coefficient can be accurately determined using $SI_{90}$. Using that, for low flow velocities, the measurement accuracy is higher than that for high velocities, in contrast to classical dynamometric methods.

From the conducted studies, it follows that the secondary flow can be used as a tool for measuring the viscosity of Newtonian liquids. The above-established relationships between the formation of secondary Dean flow in curved channels and the viscosity of the liquid open up broad prospects for further research and the development of microfluidic topologies with curved channels for measuring the rheological characteristics of liquids using the secondary flow parameters.

This concept of a device for viscosity measurement can be very useful in many microfluidic devices in which various liquids are pumped. These devices may already have pumps and flow sensors. Our viscometer can be easily and cheaply integrated into such flow-through microfluidic systems and will provide additional data on the viscosity of the fluid being pumped. Other advantages of this microfluidic mixer include a high accuracy, real-time operation, and reproducibility of results.

It should be noted that the influence of tension forces and wettability effects can be neglected. This is possible since a single-phase flow is considered, and it is assumed that the liquid completely fills the entire cross section of the microchannel. The effect of interfacial tension and wettability in microchannels is important to take into account when two-phase flows with an interface are considered.

The addition of a dye may affect the kinematic viscosity. Therefore, it is necessary to carefully select the method of flow contrasting so that it does not affect the kinematic viscosity. The addition of low molecular weight dyes that do not create a large radius hydration shell or the fluorescence of intrinsic molecules of the test liquid can be used as such methods. The choice of method depends on the specific test sample. In addition, a large number of inert dyes are known that require negligible concentrations to obtain a rich color. For example, flavin mononucleotide, chlorophyll, and its degradation products (chlorophyllide, pheophorbide a) can be used as such an inert dye.

## Figures and Tables

**Table 1:** Density and dynamic viscosity coefficient of the tested liquids at a temperature of 25 ${}^{\circ }$C.

Property	Water	30 wt%	50 wt%
Viscosity [mPa·s]	0.94 ± 0.03	2.09 ± 0.03	5.11 ± 0.03
Density [kg·m${}^{-3}$]	997	1068	1137

**Table 2:** Fitting coefficients (7), analyzed ranges of the De numbers, and the minimum angle $\theta_{min}$.

SI	$a\cdot 10^{2}$	$b\cdot 10^{2}$	$R^{2}$	AAD	MAD	De	$\theta_{\min}$
$SI_{90}$	6.787	−1.604	0.999	0.56%	1.25%	5 ÷ 20	45${}^{\circ }$
$SI_{180}$	3.414	−1.261	0.997	1.01%	2.18%	10 ÷ 30	80${}^{\circ }$
$SI_{270}$	2.843	−2.283	0.992	1.35%	3.18%	15 ÷ 35	110${}^{\circ }$
$SI_{360}$	2.874	−3.942	0.990	2.08%	4.06%	20 ÷ 45	125${}^{\circ }$

**Table 3:** Dynamic viscosity of water and aqueous solutions of glycerol.

Mass	$\mu_{\exp}$	$\mu_{\mathrm{rot}}$	$\mu_{\mathrm{ref}}$ [42]	$\varepsilon_{\exp}$	$\varepsilon_{\mathrm{rot}}$
Fraction	[mPa·s]	[mPa·s]	[mPa·s]	[%]	[%]
0	0.8897	0.94	0.8927	−0.34	5.3
0.3	2.081	2.09	2.124	−2.0	−1.1
0.5	4.864	5.11	5.004	−2.8	1.9

## Data Availability

Not applicable.
